# Intravitreal sirolimus with adjunct aflibercept versus aflibercept monotherapy for persistent, exudative age-related macular degeneration: a pilot study

**DOI:** 10.1186/s40942-022-00437-6

**Published:** 2023-01-05

**Authors:** Lucas W. Rowe, Robert J. Minturn, Lauren A. Burgett, Peter Bracha, Raj K. Maturi

**Affiliations:** 1 Department of Ophthalmology, Indiana University School of Medicine, 10300 N Illinois St, Suite 1060, Indianapolis, IN 46290 USA; 2 Department of Psychology and Neuroscience, Duke University, Durham, NC USA; 3 Wolfe Eye Clinic, West Des Moines, IA USA; 4 Midwest Eye Institute, Indianapolis, IN USA

**Keywords:** Sirolimus, Rapamycin, Aflibercept, Eylea, Anti-vascular endothelial growth factor, Exudative age-related macular degeneration

## Abstract

**Background:**

To determine the safety and efficacy of intravitreal sirolimus and adjunct aflibercept in subjects with persistent, exudative age-related macular degeneration despite previous intravitreal anti-vascular endothelial growth factor (VEGF) treatment.

**Methods:**

This institutional review board approved, registered (NCT02732899), prospective, subject-masked, single center, randomized controlled trial in subjects with persistent, exudative age-related macular degeneration compared alternating monthly intravitreal sirolimus and aflibercept (combination) versus aflibercept monotherapy (control) every 2 months over the course of 36 weeks. The primary measure of efficacy in the study was the mean change in central subfield thickness.

**Results:**

20 subjects were enrolled in the study, with 10 subjects assigned to each treatment group. Subjects had an average of 38 previous anti-VEGF injections. Mean central subfield thickness decreased in the combination group by 54.0 μm compared to 0.1 μm in the control group (*p* = *0.28*). Mean visual acuity improved in the combination group by 2.5 ETDRS letters versus 0.8 ETDRS letters in the control group (*p* = *0.42*). There were no serious ocular adverse events in either group; however, there were three serious systemic events in the combination group, including hospitalizations due to pancreatitis, pneumonia, and worsening hypertension.

**Conclusion:**

There was no statistically significant difference in the mean central subfield thickness change between the combination and control groups. However, intravitreal sirolimus with adjunct aflibercept did appear to have potential anatomical benefits as a treatment for persistent, exudative age-related macular degeneration and requires further investigation with a larger cohort to better understand the potential risks and benefits.

*Trial registration:* ClinicalTrials.gov, NCT02732899. Registered 11 March 2016, https://clinicaltrials.gov/ct2/show/NCT02732899. This trial was approved by the institutional review board at Advarra. Funding was provided by an investigator-initiated grant from Santen. Santen played no role in the design or implementation of this study.

## Background and objective

The Comparison of Age-Related Macular Degeneration Treatment Trials (CATT) revealed that despite the success of chronic anti-vascular endothelial growth factor (VEGF) for age-related macular degeneration (AMD), there remained limitations associated with long term use of the therapy. At 5 years, 61% of patients demonstrated persistent, intraretinal fluid, which correlates with worse visual acuity outcomes [1, 2]. In addition, the Inhibition of VEGF in Age-related choroidal Neovascularization (IVAN) trial demonstrated that monthly anti-VEGF treatments are associated with a higher risk and greater development of geographic atrophy (GA) [3]. Increase in GA and total lesion size were strongly associated with greater decrease in visual acuity [2]. These findings suggest the need for alternative or adjuvant therapeutic options for persistent, exudative AMD in order to provide an alternative treatment and mitigate potential development of GA.

Sirolimus, also known as rapamycin, is a macrolide produced by the bacterium *Streptomyces hygroscopicus.* This compound inhibits the mammalian target of rapamycin (mTOR)*.* The mTOR kinase pathway is involved in the regulation of immune responses, T-cell proliferation, and proinflammatory cytokine production. The FDA has approved the use of sirolimus in kidney transplant rejection and coronary stent coating due to these immune modulating properties. Recently, sirolimus has undergone a successful phase III trial and is under FDA consideration for the treatment of noninfectious uveitis [4–6]. In addition to its immunosuppressive qualities, sirolimus has demonstrated anti-angiogenic properties due to mTOR’s role in the VEGF cascade, prompting a pilot study investigating the use of sirolimus for persistent, exudative AMD [7, 8]. Sirolimus has been evaluated in numerous preclinical AMD models, demonstrating reduction of choroidal neovascularization (CNV) when administered systemically or subcutaneously [9, 10]. Furthermore, intravitreal sirolimus was well-tolerated in rabbits and revealed retino-choroidal migration supporting its potential use in chorioretinal disease [11]. A recent pilot study by Minturn et al. compared intravitreal sirolimus monotherapy (every 2 months) versus monthly anti-VEGF for persistent, exudative AMD. The study showed a statistically significant decrease in central subfield thickness (CST) in subjects treated with sirolimus, but reported a higher number of serious ocular adverse events when compared to the anti-VEGF group [12]. The objective of this study is to investigate the safety and efficacy of intravitreal sirolimus and adjunct aflibercept therapy versus aflibercept monotherapy.

## Methods

### Study design

This prospective, 36-week, subject-masked, phase 2 trial was conducted at a single site with the goal of evaluating the safety and efficacy of sirolimus with adjuvant aflibercept in patients with persistent, exudative AMD. Each subject provided written informed consent before enrollment. The study site complied with the Health Insurance Portability and Accountability Act and adhered to the tenets set out in the 1964 Declaration of Helsinki.

### Participants

Eligible patients were recruited from the practice of the principal investigator. Inclusion criteria was defined as best corrected visual acuity (BCVA) between 5 and 75 Early Treatment of Diabetic Retinopathy Study (ETDRS) letters, presence of CNV secondary to AMD, at least 3 previous intravitreal anti-VEGF injections in the past 6 months, and a lack of response to anti-VEGF therapy defined as continued subretinal or intraretinal fluid with a decrease in central subfield thickness of less than 100 μm since the last injection. Only one eye was evaluated in each patient, and if both eyes were eligible for inclusion, the eye with greater fluid was generally chosen. Exclusion criteria was met if the study eye had a greater than 100 μm decrease in central subfield thickness on ocular coherence tomography (OCT) from the last standard of care visit, major ophthalmic surgery within the past 3 months, any ophthalmic surgery in the past 90 days, significant ocular disease, diagnosis other than exudative AMD, significant epiretinal membrane, significant vitreoretinal traction, or hypersensitivity to components of study medication.

### Treatment and follow-up

The first subject was enrolled on April 12th, 2016, and the last subject completed the study on March 20th, 2017. Subjects were randomized in a 1:1 ratio to a combination group receiving sirolimus (440 μg/20 μL) every 8 weeks, and aflibercept (EYLEA^®^, 2 mg/0.05 mL) 4 weeks after the sirolimus, as per the schedule below (Table 1). The aflibercept group (control) received this drug every 8 weeks with a sham treatment to maintain subject masking. Subjects in the treatment group were given an additional dose of sirolimus at week 4 as a loading dose (Table 1). Dosing of sirolimus was formulated based on previous uveitis trials and pharmacokinetics of intravitreal sirolimus [5, 6, 13, 14]. At weeks 24 and 32, all subjects whose VA and CST had not worsened by greater than or equal to 5 ETDRS letters or 50 μm were deferred treatment and given sham injections.

**Table 1:** Treatment Schedule

Group	Visit (Week)										
	Baseline	1	4	8	12	16	20	24	28	32	36
Treatment group (sirolimus + aflibercept)	S	A	S	A	S	A	S	A^a^	S	A^a^	Final Exam
Control group (aflibercept monotherapy)	A	SH	SH	A	SH	A	SH	A^a^	SH	A^a^	Final Exam

The treatment group was treated with sirolimus (S) and adjunct aflibercept (A). The control group received aflibercept (A) or a sham (SH) treatment

^a^Indicates that treatment is at the discretion of the investigator based on meeting adequate anatomical and visual acuity benchmarks

### Safety measures and assessments

All injections followed a standardized aseptic injection protocol including use of sterile equipment, topical anesthetic, and topical antimicrobial. All sham injections were done by placing the tip of the syringe without any needle against the conjunctiva. Subjects were asked at each visit about any recent changes of note or hospitalizations. Any concerns were documented with date, duration, need for hospitalization, and need for intervention. Primary safety endpoints included minor adverse ocular events, minor adverse systemic events, serious adverse ocular events, and serious adverse systemic events.

Rescue criteria for all subjects included a decrease of 10 or more ETDRS letters at two consecutive visits, a decrease of 15 or more ETDRS letters at any singular visit, an increase of CST greater than or equal to 50 μm with a decrease of 5 ETDRS letters, the presence of new or worsening hemorrhage, new extrafoveal fluid, or at the discretion of the investigator. If any of the rescue criteria were met, the subject was immediately treated with an anti-VEGF injection (aflibercept), the standard of care for exudative AMD if the subject was to receive either sham or sirolimus at that visit.

### End points and statistical analysis

Change in central subfield thickness between baseline and week 36 was chosen as the primary endpoint. Change in CST was chosen due to the challenging nature of persistent fluid in this patient population, for which a change in visual acuity would be less likely. Secondary measures included changes in BCVA, intraretinal fluid (IRF) volume, subretinal fluid (SRF) volume, and central 1 mm foveal volume. The same Heidelberg Spectralis machine was used to obtain both images and CST. Prior to data analysis, the automated CST segmentation was verified and manually adjusted in an anonymized manner, as necessary. The central 1 mm foveal volume (mm^3^), subretinal fluid volume (mm^3^), and intraretinal fluid volume (mm^3^) were calculated by an independent, masked OCT reader. Due to the small sample size, analysis was limited to two-sample t-tests with unequal variance using Stata (version 16, StataCorp LLC, College Station, TX, USA).

## Results

### Demographics and baseline characteristics

20 eyes were enrolled in the study, with 10 eyes assigned to each treatment group. The first subject was enrolled on April 12th, 2016, and the last subject completed the study on March 20th, 2017. The baseline characteristics of each group are summarized in Table 2, there was no significant difference in any baseline characteristic. The 36-week evaluation was performed in 100% of eyes in both groups. There was only one missed visit in the entire study, yielding a 99% compliance rate.

**Table 2:** Baseline subject characteristics

Demographic	Treatment group (sirolimus + aflibercept) (N = 10)		Control group (aflibercept monotherapy) (N = 10)		p-value
	Mean	SD	Mean	SD	
Age at baseline (years)	80.8	7.2	78.4	9.7	0.54
Intraocular pressure (mm Hg)	16.3	2.9	14.3	2.0	0.09
Duration of exudative AMD (months)	64.2	26.1	67.7	56.4	0.86
Previous intravitreal anti-VEGF injections (# of shots)	43.0	18.1	34.1	21.7	0.33
Baseline visual acuity (ETDRS letters)	62.6	9.0	61.0	14.1	0.77
Baseline central subfield thickness (μm)	444.2	114.8	507.9	118.0	0.24
Baseline subretinal volume (mm^3^)	11.0	26.8	9.8	14.4	0.91
Baseline intraretinal volume (mm^3^)	0.4	1.3	1.3	3.7	0.52
Central 1 mm foveal volume (mm^3^)	8.5	0.8	9.3	1.0	0.11
Weeks since last anti-VEGF treatment	4.8	0.6	6.3	2.5	0.10

This table summarizes the two study populations at the time of enrollment in the study. There was no statistically significant difference between our two study groups in any of the measured variables

In the treatment group, ten eyes (100%) attended all exam visits and received full dosing of sirolimus. At week 24, two subjects (20%) met the criteria for deferral of treatment with aflibercept, while at week 32, four subjects (40%) met the criteria for deferral of treatment. In the control group, nine eyes (90%) attended all exam visits. The only missed visit by a subject occurred at week 32 in the control group and was deemed to be non-related to the subject’s ocular disease or treatment. No subjects in the control group met deferral of treatment criteria at any point during this trial. In addition, no subject met the rescue criteria during the duration of this trial.

### Efficacy

**Fig. 1:**
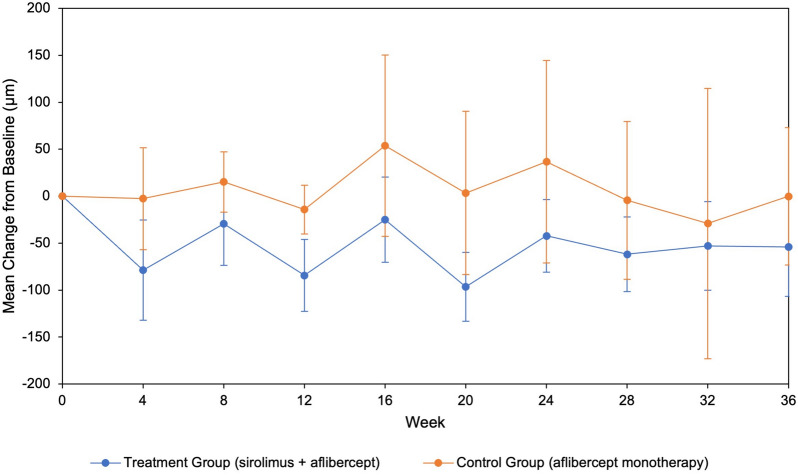
Mean change in CST from baseline (95% C.I.). This graph depicts the change in central subfield thickness from baseline to 36-week visit. The average change from baseline for the treatment group was − 54.0 μm (− 106.7, − 1.4). The average change from baseline for the control group was − 0.1 μm (− 73.1, 72.9)

**Fig. 2:**
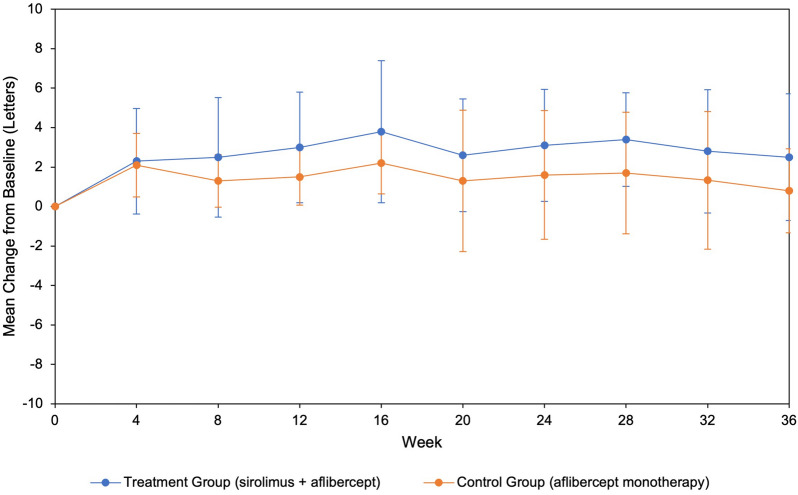
Mean change in BCVA from baseline (95% C.I.). This graph depicts the change in BCVA from baseline to 36-week visit. The average change from baseline for the treatment group was 2.5 letters (− 0.7, 5.7). The average change from baseline for the control group was 0.8 letters (− 1.3, 2.9)

The central subfield thickness outcomes are summarized in Fig. 1. At 36 weeks, mean central subfield thickness decreased by 53.9 μm more in the treatment group than the control group; however, there was no statistically significant difference (*p* = *0.28*). The percentage decrease in mean central subfield thickness was 12.0% in the treatment group versus 2.5% in the control group (*p* = *0.30*). The 36-week visual acuity outcomes are demonstrated in Fig. 2. At 36 weeks, the mean BCVA change was improvement of 2.5 ETDRS letters in the treatment group and improvement of 0.8 letters in the control group (*p* = *0.42*). BCVA percentage increase was 4.7% in the treatment group and 2.2% in control group (*p* = *0.56*). At 36 weeks, the mean change in subretinal fluid (*p* = *0.54*), intraretinal fluid (*p* = *0.60*), and central 1 mm foveal volume (*p* = *0.78*) were not found to be statistically significant between the two groups (Table 3).

**Table 3:** Visual acuity and macular thickness measurements

	Treatment group (sirolimus + aflibercept)	Control group (aflibercept monotherapy)	p-value
BCVA Change (ETRDS Letters)	2.5	0.8	0.42
Central Subfield Thickness (μm)	− 54.0	− 0.1	0.28
Subretinal Fluid (N)	9	9	–
SRF Change (mm^3^)	− 9.32	− 3.02	0.54
Intraretinal Fluid (N)	4	4	–
IRF Change (mm^3^)	− 0.43	− 0.99	0.60
Corrected IRF Change (mm^3^) ^a^	− 1.02	− 2.48	0.59
Central 1 mm (N)	10	10	–
Foveal Change (mm^3^)	− 0.49	− 0.66	0.78

^a^Subjects who did not have intraretinal fluid during the course of the study were excluded from this calculation to gain a better appreciation of the true change in IRF

**Fig. 3:**
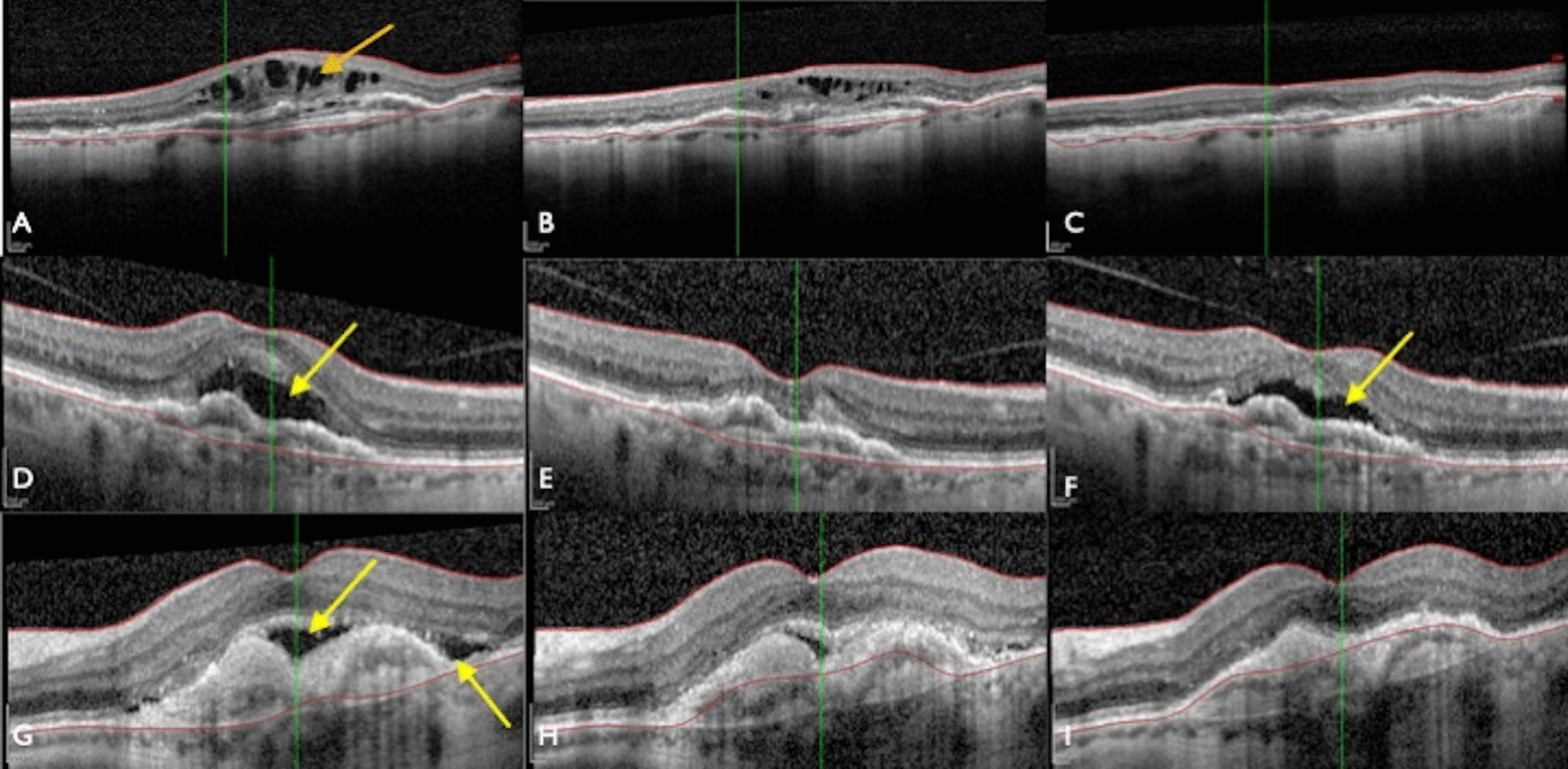
Anatomical changes in three subjects treated with intravitreal sirolimus and adjunct aflibercept. Image A shows several pockets of intraretinal fluid (orange arrow), which dissipate by week 36 (image C). Images D and G show several pockets of subretinal fluid (yellow arrows) which decrease in volume over the course of treatment

A total of nine subjects in the treatment group had subretinal fluid at baseline, with two (22.2%) experiencing complete resolution of subretinal fluid. In contrast, ten subjects in the control group had subretinal fluid at baseline with three (30.0%) experiencing complete resolution. A total of three subjects in the treatment group had intraretinal fluid at baseline, with two (66.7%) experiencing complete resolution of intraretinal fluid. In comparison, three subjects in the control group had intraretinal fluid at baseline with one (33.3%) experiencing complete resolution at 36 weeks (Fig. 3).

### Safety

The safety outcomes for the two groups are summarized in Table 4. No subjects developed serious ocular adverse events during this study. There were three documented serious systemic adverse events in the treatment group: pancreatitis, pneumonia, and worsening hypertension. No subjects developed systemic adverse events in the control group. The average intraocular pressure (IOP) at week 36 was 15.6 mmHg in the treatment group and 13.4 mmHg in the control group at conclusion of the study. The average change in IOP at all visits showed no statistical significance between groups.

**Table 4:** Systemic and ocular adverse events

	Treatment group (sirolimus + aflibercept)	Control group (aflibercept monotherapy)
Systemic events		
Serious adverse events	3^a^	0
Minor adverse events	4	2
Ocular events		
Serious adverse events	0	0
Minor adverse events	4	2

This table lists the number of adverse events in each group, separated into systemic and ocular adverse events

^a^ Hospitalizations due to pancreatitis, pneumonia, and worsening hypertension. All serious adverse events resolved without sequelae

## Discussion

Currently, the main treatment modality for exudative AMD is intravitreal anti-VEGF agents. In this study, we presented a small cohort of subjects treated with the mTOR inhibitor sirolimus and adjunct aflibercept. Previously, Minturn et al. noted a significant decrease in CST demonstrating potential benefits of sirolimus in persistent, exudative AMD; however, the study was limited by small cohort size, safety concerns, and the need for some subjects to be rescued from sirolimus to anti-VEGF [12]. This current study was designed to investigate the safety and efficacy of sirolimus with adjuvant aflibercept in patients with persistent, exudative AMD.

Results of this trial suggest anatomical benefits for subjects treated with sirolimus and adjunct aflibercept; however, the results lacked statistical significance which may be due to a small sample size. Regarding CST, the primary outcome, a trend towards better drying in the combination treatment group was observed; with this cohort demonstrating 54 μm more thinning than subjects in the control cohort (*p* = *0.28*). These results corroborate those ascertained in the study by Minturn et al., where the sirolimus monotherapy group demonstrated 60 μm more thinning compared to the anti-VEGF group [12]. Although also limited by a small sample size, the previous study enrolled 17 patients in the treatment group and 20 patients in the control group, which added more statistical power in comparison to the current study and may have facilitated the statistical significance of the results. It is also noteworthy that the control group in the previous study received aflibercept or bevacizumab whereas the current study included aflibercept only. Comparatively, the CATT trials included ranibizumab or bevacizumab and revealed similar anatomical outcomes between the drugs. [1]

In addition, subjects showed a decrease in the secondary measures of SRF and IRF, but these too lacked statistical significance. Nonetheless, there were clear anatomical benefits in individual combination subjects that were not noted in the control group. In Fig. 3, we show the presence of intraretinal fluid and subretinal fluid over the course of the 36-week treatment cycle. Complete resolution of subretinal fluid occurred in 22% of the treatment group versus 30% of the control group. Comparatively, complete resolution of intraretinal fluid occurred in 67% of the treatment group and 33% of the control group. Given the anti-angiogenic effects of sirolimus, the decrease in subretinal and intraretinal fluid could be secondary to the decreased angiogenesis similar to effects found in anti-VEGF treatments [7, 9, 15, 16]. Despite the very small sample size, the use of intravitreal sirolimus and adjunct aflibercept displayed an encouraging ability to decrease or eliminate intraretinal fluid, a potential positive prognosticator in patients with persistent, exudative AMD.

Previously, intravitreal sirolimus monotherapy for persistent, exudative AMD raised concern for a higher rate of severe ocular adverse events, including uveitis, retinal artery occlusion, and subretinal hemorrhage [12]. Similarly, in trials for geographic atrophy and posterior uveitis, intravitreal sirolimus was associated with the development of anterior uveitis [5, 17]. In contrast to these prior studies, no significant ocular adverse events were observed throughout the course of this study, including those in the treatment group who received a loading dose of sirolimus at baseline and week 4. However, there were more systemic adverse events in the treatment group. Traditionally, common side effects of systemic sirolimus administration include anemia, nephrotoxicity, mucocutaneous ulcers, and joint pain [18]. A pharmacokinetic study involving intravitreal sirolimus demonstrated systemic exposure is minimal [19], and previous studies investigating intravitreal sirolimus found a low incidence of non-ocular adverse events [4–6, 12, 17]. It is difficult to ascertain whether the lack of severe ocular adverse events or increased number of severe systemic adverse events were related to sirolimus, due to the small sample size, or the general health of the subjects enrolled in this study.

Several limitations of this study include a small sample size, short duration of follow-up, and variability in treatment history. We also did not collect past systemic medical history on the subjects and, therefore, it was difficult to elucidate whether the differences in systemic adverse events were influenced by the general health of the subjects. In addition, we chose a dose of sirolimus based on previous posterior uveitis trials, and without a prior dose escalation study for exudative AMD, the ideal dose of sirolimus is currently unknown. Furthermore, the optimal frequency of sirolimus and anti-VEGF injections as dual therapy is currently unknown. It is possible that subjects in this study did not receive frequent enough aflibercept injections. This is supported by the fluctuating central subfield thickness averages seen in Fig. 1. A similar trend was seen among patients treated with aflibercept every eight weeks in the “VEGF Trap-Eye: Investigation of Efficacy and Safety in Wet AMD” studies (VIEW 1 and VIEW 2), which was concluded to be secondary to inadequate frequency of administration [20]. It is possible, given the severity of AMD in our target population, that higher dosages or greater frequency of intravitreal sirolimus and aflibercept could be necessary for optimal treatment efficacy.

## Conclusion

Persistent, exudative AMD with subretinal or intraretinal fluid can be a challenge to treat in patients despite adequate anti-VEGF therapy. In this study, we did not find a statistically significant decrease of central subfield thickness, IRF, or SRF after treatment with sirolimus and adjunct aflibercept in comparison to aflibercept monotherapy. However, the combination therapy virtually eliminated the presence of intraretinal fluid present at baseline in several subjects, despite a long duration of previous treatment with anti-VEGF agents. This pilot study is limited due to the small sample size and lack of previous dosing studies for the use of sirolimus in exudative AMD. Based on our results, intravitreal sirolimus with adjunct aflibercept appears to have potential anatomical benefits; however, dual therapy requires further investigation to better understand the risks and benefits for its use in persistent, exudative macular degeneration.

## Data Availability

The datasets used and analyzed during the current study are available from the corresponding author on reasonable request.

## Abbreviations

AMD: Age-related macular degeneration
BCVA: Best corrected visual acuity
CATT: Comparison of age-related macular degeneration trial
CMT: Central macular thickness
CNV: Choroidal neovascularization
CST: Central subfield thickness
ETDRS: Early treatment of diabetic retinopathy study
GA: Geographic atrophy
IOP: Intraocular pressure
IRF: Intraretinal fluid
mTOR: Mammalian target of rapamycin
OCT: Ocular coherence tomography
SRF: Subretinal fluid
VEGF: Vascular endothelial growth facto**r**

## References

[1] Maguire MG, Martin DF, Ying GS, Jaffe GJ, Daniel E, Grunwald JE (2016). Five-Year outcomes with anti-vascular endothelial growth factor treatment of neovascular age-related macular degeneration: the comparison of age-related macular degeneration treatments trials. Ophthalmology.

[2] Sharma S, Toth CA, Daniel E, Grunwald JE, Maguire MG, Ying GS (2016). Macular morphology and visual acuity in the second year of the comparison of age-related macular degeneration treatments trials. Ophthalmology.

[3] Chakravarthy U, Harding SP, Rogers CA, Downes SM, Lotery AJ, Culliford LA (2013). Alternative treatments to inhibit VEGF in age-related choroidal neovascularisation: 2-year findings of the IVAN randomised controlled trial. Lancet Lond Engl.

[4] Nguyen QD, Merrill PT, Sepah YJ, Ibrahim MA, Banker A, Leonardi A (2018). Intravitreal sirolimus for the treatment of noninfectious uveitis: evolution through preclinical and clinical studies. Ophthalmology.

[5] Nguyen QD, Merrill PT, Clark WL, Banker AS, Fardeau C, Franco P (2016). Intravitreal sirolimus for noninfectious uveitis: a phase III sirolimus study assessing double-masked uveitis treatment (SAKURA). Ophthalmology.

[6] Merrill PT, Clark WL, Banker AS, Fardeau C, Franco P, LeHoang P (2020). Efficacy and safety of intravitreal sirolimus for noninfectious uveitis of the posterior segment: results from the sirolimus study assessing double-masked uveitis treatment (SAKURA) program. Ophthalmology.

[7] Nakahara T, Morita A, Yagasaki R, Mori A, Sakamoto K (2017). Mammalian target of rapamycin (mTOR) as a potential therapeutic target in pathological ocular angiogenesis. Biol Pharm Bull.

[8] Nussenblatt RB, Byrnes G, Sen HN, Yeh S, Faia L, Meyerle C (2010). A randomized pilot study of systemic immunosuppression in the treatment of age-related macular degeneration with choroidal neovascularization. Retina.

[9] Dejneka NS, Kuroki AM, Fosnot J, Tang W, Tolentino MJ, Bennett J (2004). Systemic rapamycin inhibits retinal and choroidal neovascularization in mice. Mol Vis.

[10] Yagasaki R, Nakahara T, Ushikubo H, Mori A, Sakamoto K, Ishii K (2014). Anti-angiogenic effects of mammalian target of rapamycin inhibitors in a mouse model of oxygen-induced retinopathy. Biol Pharm Bull.

[11] de Manzano ARP, Peyman GA, Khan P, Kivilcim M, Chevez-Barrios P, Takahashi W (2009). Testing intravitreal toxicity of rapamycin in rabbit eyes. Arq Bras Oftalmol.

[12] Minturn RJ, Bracha P, Klein MJ, Chhablani J, Harless AM, Maturi RK (2021). Intravitreal sirolimus for persistent, exudative age-related macular degeneration: a pilot study. Int J Retina Vitr.

[13] Nguyen QD, Merrill P, Clark WL (2017). Efficacy and safety results from the SAKURA program: two phase III studies of intravitreal sirolimus every other month for non-infectious uveitis of the posterior segment. Invest Ophthalmol Vis Sci.

[14] Mudumba S, Bezwada P, Takanaga H, Hosoi K, Tsuboi T, Ueda K (2012). Tolerability and pharmacokinetics of intravitreal sirolimus. J Ocul Pharmacol Ther.

[15] Karar J, Maity A (2011). PI3K/AKT/mTOR pathway in angiogenesis. Front Mol Neurosci.

[16] Guba M, von Breitenbuch P, Steinbauer M, Koehl G, Flegel S, Hornung M (2002). Rapamycin inhibits primary and metastatic tumor growth by antiangiogenesis: involvement of vascular endothelial growth factor. Nat Med.

[17] Gensler G, Clemons TE, Domalpally A, Danis RP, Blodi B, Wells J (2018). Treatment of geographic atrophy with intravitreal sirolimus: the age-related eye disease study 2 ancillary study. Ophthalmol Retina.

[18] Stallone G, Infante B, Grandaliano G, Gesualdo L (2009). Management of side effects of sirolimus therapy. Transplantation.

[19] Chu D, Mudumba S (2019). Pharmacokinetics of intravitreal sirolimus in a subset of subjects with non-infectious uveitis of the posterior segment of the eye. Invest Ophthalmol Vis Sci.

[20] Heier JS, Brown DM, Chong V, Korobelnik JF, Kaiser PK, Nguyen QD (2012). Intravitreal aflibercept (VEGF Trap-Eye) in wet age-related macular degeneration. Ophthalmology.

